# Instrument to evaluate the perception of minimal contrasts in Chilean Sign Language – reliability evidence

**DOI:** 10.1590/2317-1782/20232022184en

**Published:** 2023-12-04

**Authors:** Carlos González Herrera, Themis Maria Kessler, Karina Carlesso Pagliarin

**Affiliations:** 1 Departamento de Ciencias de la Fonoaudiología, Facultad de Ciencias de la Salud, Universidad de Talca – UTALCA - Talca (VII Región del Maule), Chile.; 2 Programa de Pós-graduação em Distúrbios da Comunicação Humana, Universidade Federal de Santa Maria – UFSM - Santa Maria (RS), Brasil.

**Keywords:** Evaluation, Sign Language, Reliability, Child, Deafness

## Abstract

**Purpose:**

Obtain evidence of the test reliability to evaluate the perception of minimum contrasts in Chilean Sign Language (LSCh).

**Methods:**

Ten deaf children and adolescents aged between 7 and 14 years participated in this study. They were evaluated with the test of perception of minimal contrasts in LSCh. The test was reapplied 11 and 14 days after the first application (test-retest reliability). Spearman's Rho correlation was performed. During the first application, authorization was requested from the parents of the children and adolescents to record the responses of the participants so that another evaluator could re-score the protocols, in order to obtain inter-rater reliability. *First-order agreement coefficient* (AC1) Gwet’s was used for data analysis.

**Results:**

Test-retest obtained a strong and significant correlation (Rho= 0.741; p=0.014). The concordance values obtained inter-rater vary between 0.962 and 1 (p<0.001), indicating that the test presents almost perfect concordance.

**Conclusion:**

The minimum pairs perception test in LSCh presents satisfactory test-retest and inter-rater reliability.

## INTRODUCTION

Deaf people are a heterogeneous population^(1)^. Both children and adolescents have variability in sign language proficiency^(2)^. Sometimes, sign language is not the first language to which the child is exposed, which represents a great challenge when it comes to evaluation. Approximately, 90-95% of deaf children have hearing parents who do not know sign language, which often delays the child's linguistic development since the priority is the acquisition of speech^(3)^. On the other hand, deaf children of deaf parents have contact with sign language from birth and therefore a greater possibility of acquiring sign language from an early age.

In recent years, there has been great interest by researchers in the creation of instruments that measure the development of visual language in deaf children^(4)^. The evaluation of a child's language requires adequate measures that are appropriate to their age, language, and culture^(5)^. There are few valid and reliable evaluations available for deaf children, especially those that evaluate language among deaf children in their first 5 years of life^(6,7)^. In addition, a small number of evaluations are listed as available to measure the developmental skills of sign language in preschool children^(4)^. The above contrasts with the growing interest in the evaluation of language disorders in deaf children who use sign language as their first language^(4,8)^. Many language assessment instruments developed for deaf people are adaptations of instruments made to assess language in hearing people. In this way, they do not reflect the real needs of the deaf person^(7)^. It is possible to identify some reasons for the scarcity of these instruments, such as the heterogeneity of the population and sign language, and the low access to native deaf people^(9)^.

Currently, in Chilean reality, evaluation instruments built for hearing people are used to evaluate deaf people. For this reason, the construction of appropriate tests is required to evaluate language in the sign language (visuospatial) modality.

There are tests with psychometric properties but they are in other languages not in Spanish, for example: the British Sign Language Vocabulary Tasks^(10)^; the American Sign Language Sentence Repetition Test^(11)^; The Visual Communication and Sign Language Checklist^(12)^. Also, in Brazilian Sign Language (LIBRAS), an instrument was developed to evaluate the mastery of minimal contrasts^(13)^ which serves to know the performance of deaf children in the recognition and perception of minimal contrast pairs in LIBRAS. Furthermore, the development of the perception of minimal contrasts in LIBRAS was studied in hearing children of deaf parents^(14)^.

The evaluation of the minimum contrasts allows us to know the perception of children and adolescents of the different training parameters of the language. Furthermore, it allows us to verify some aspects of the development of these parameters in children^(13)^, which can influence communication as a whole. It is important to time exposure to sign language since it makes sense in the evaluation of the perception of minimal contrasts in LIBRAS. The longer the time of exposure to sign language, the better the performance in the perception of minimum contrasts^(14)^.

The minimum contrasts perception evaluation test was adapted to Chilean Sign Language (LSCh)^(15)^ and is based on Vargas et al.^(13)^. It consists of 24 items distributed as follows: 7 for Handshape, 5 for location, 8 for movement and 4 for orientation. Among the 24 pairs, 2 items were selected as examples (January-February and young-suffer). The evaluation went through a content validation process in 7 stages: 1. Collection of minimal contrast pairs in LSCh; 2. Analysis by specialist judges (two native deaf people and two hearing people who are LSCh interpreters); 3. Minimum contrast pair design; 4. Analysis of non-specialist judges (six deaf participants between 7 and 14 years old); 5. Test preparation; 6. Video recording; 7. Pilot Study. These stages allowed us to present satisfactory content validity.

Despite being a substantial advance and becoming the first test to evaluate minimal contrasts in Chilean sign language and having good validity indices, more reliable psychometric studies are needed. From that, this research aims to obtain evidence of the reliability of the minimum contrasts’ perception test in LSCh.

## METHOD

This study is linked to a research project duly registered and approved by the Research Ethics Committee of the Federal University of Santa María, RS, Brazil, page number 3,022,041. Authorization was requested from those responsible for the children and adolescents who participated in the research following the standards determined by the National Health Council in its resolution 510/16 through the signing of the Informed Consent Form (ICF).

### Participants

Ten deaf children and adolescents who attend a special school for deaf people and who do not have any other diagnosis participated in this study. There were four boys and six girls between 7 and 14 years old at the time of the evaluation (M=11.6 years). The participants were recruited by non-probabilistic convenience sampling, all of whom were from the city of Talca, Chile. The inclusion criteria of this research for children and adolescents were being an LSCh user, between 6 and 18 years old, and having a diagnosis of severe or profound bilateral hearing loss. The exclusion criteria were having some other type of observable deficit (neurological injury, syndromes, uncorrected visual deficit). Data were collected through an interview which was applied to the parents or caregivers of children and adolescents participating in this study. The interview asked about the age of exposure to sign language by children and adolescents, and the sample average of 5 years. Finally, two speech-language therapist evaluators, LSCh interpreters recognized by the Ministry of Education, were recruited for inter-rater analysis.

### Test-retest reliability

To establish test-retest reliability, the minimum contrasts perception test in LSCh was fully applied to 10 deaf children and adolescents at two different times. The time between the test and the retest varied between 11 and 14 days.

The evaluator in charge showed the booklet containing the images of the minimal contrast pairs to deaf children and adolescents. Additionally, he showed them a video where the instructions in LSCh were delivered by an interpreter. The deaf child or adolescent were instructed to watch the video, understand the instructions given, and answer which is the pair of minimum contrast that the interpreter represented, indicating the chosen alternative by pointing his or her finger in the booklet. Correct answers were assigned a score of 1 and incorrect answers received a score of 0.

For data analysis, Spearman's Rho correlation coefficient was used, correlating the total score of the test with the score of the retest. Values between 0.31 and 0.5 were considered weak; between 0.51 and 0.7 moderate; between 0.71 and 0.9 strong, and greater than 0.9 very strong^(16)^.

### Interrater reliability

During the first application of the minimum contrast perception test in LSCh to the 10 deaf children and adolescents, we requested permission from the parents to record the procedure. We used a professional camera, and the responses of the 10 deaf children and adolescents were recorded. The moment in which they wrote down the answer on the paper sheet was recorded and after a month, another evaluator reanalyzed the videos and rescored the protocols. To verify the agreement between the judges, we considered the statistical calculation first-order agreement coefficient (AC1) of Gwet^(17)^.

## RESULTS

Table 1 shows the descriptive data obtained in the test-retest analysis considering the training parameter of the items and the correlation test.

**Table 1:** Test–retest correlation analysis

**Item**	**Training parameter**	**Test M(SD)**	**Retest M(SD)**	**Rho**	**p-value**
Cheese – celebrate	Handshape/6	5.6 (0.70)	5.5 (0.71)	0.741	0.014
Wednesday – Friday					
Turtle – Snail					
Cow - Bull					
Tree – December					
Duck – Chicken					
Notebook – Book	Movement/7	5.2 (1.40)	6.6 (0.70)		
Bee – Fly					
Backpack – Jacket					
Pencil – Gun					
Llama – Giraffe					
Near - far					
Walk – Jump					
Example – sign language	Orientation/4	3.4 (0.97)	3.7 (0.48)		
Thursday – Play					
How? - Busy					
Positive – Negative					
Nice – Son	location/5	3.7 (1.25)	4.6 (0.70)		
Wash – Caress					
Butter – Paint					
Orange - fruit					
Yellow-green					

**Caption:** M = Media; SD = Standart desviation.

The results of the test-retest reliability and the correlation coefficient were strong, positive, and significant (Table 1). A better score is observed for children in the retest. Table 2 shows the inter-rater reliability results for each training parameter.

**Table 2:** Interrater reliability results

**Training parameter**	**Gwet's AC1**	**p-value**
Handshape/6	0.962	<0.001
Movement/7	0.977	<0.001
Orientation/ 4	0.966	<0.001
Location/5	1	<0.001

Table 2 shows that the agreement values obtained vary between 0.962 to 1 (p<0.001), indicating that the test has almost perfect agreement.

## DISCUSSION

The evaluation of language in the visuospatial modality (sign language) has become a challenge for researchers, evaluators, and clinicians^(1)^. Few tests have psychometric properties (reliable and valid) to evaluate deaf children who communicate through sign language^(7)^. From that, the objective of this research was to obtain evidence of the reliability of the minimum contrast perception test in the LSCh.

In this investigation, satisfactory reliability results were observed for the instrument to Evaluate the Perception of Minimum Contrasts in Chilean Sign Language. Reliability is one of the main quality criteria of an instrument since it means that it is capable of reproducing a result stably over time or from different examiners^(18,19)^. Also, the instrument managed to go through a rigorous adaptation and validation process, where linguistic and cultural characteristics and differences were considered for adaptation to the LSCh^(20)^.

The test-retest reliability showed a strong association between the results of the test and those of the retest, which demonstrates the temporal stability of the test^(19,21)^. Better performance of children is observed in the retests. Such a result may be related to learning/memory since the retest was carried out in a short period ^(18)^, or even to the acquisition of the training parameter. The inter-rater analysis (Table 2) shows almost perfect agreement, indicating precision when it involves different examiners of the same subject.

The results obtained in this research are a contribution to the Chilean deaf community, as it becomes the first instrument to evaluate minimal contrasts in visuospatial modality (sign language) with validity and reliability. However, more studies of construct validity, criterion validity, sensitivity, and specificity are necessary.

Understanding how deaf children and adolescents perceive minimal contrasts can help with aspects such as the clinician's understanding of how they are acquiring, understanding, and executing signs. The perception of minimal contrasts that change the composition of a sign is important for the acquisition of sign language^(13)^. Therefore, the instrument investigated here is of utmost importance for people who research this population.

As a limitation of this research, we observed that the low number of participants was because it was carried out in the context of the COVID-19 pandemic, and the sanitary conditions in Chile at that time were at critical levels. This is why we recommend that future studies expand the sample of both deaf children and adolescents and judges.

## CONCLUSION

The test to evaluate the perception of minimal contrasts in LSCh has good test-retest reliability indices and is accurate for analysis involving different evaluators, presenting satisfactory inter-rater reliability. It is a replicable and consistent instrument, that is, reapplication on the same subject produces similar results.
