# Supplementary material for: Instrument to evaluate the perception of minimal contrasts in Chilean Sign Language – reliability evidence
Source: Codas. 2023 Dec 4;35(6):e20220184. doi: 10.1590/2317-1782/20232022184en (PMC10750821; doi:10.1590/2317-1782/20232022184en)
Supplement: Translation

# Translation: Instrumento de evaluación de la percepción de los contrastes mínimos en Lengua de Señas Chilena - evidencias de confiabilidad

**DOI:** 10.1590/2317-1782/20232022184es

**Authors:** Carlos González Herrera, Themis Maria Kessler, Karina Carlesso Pagliarin

**Affiliations:** 1 Departamento de Ciencias de la Fonoaudiología, Facultad de Ciencias de la Salud, Universidad de Talca - UTALCA - Talca (VII Región del Maule), Chile.; 2 Programa de Pós-graduação em Distúrbios da Comunicação Humana, Universidade Federal de Santa Maria - UFSM - Santa Maria (RS), Brasil.

**Keywords:** es: Evaluación, Lengua de Señas, Confiabilidad, Sordera, Niño

## Abstract

**Objetivo:**

Obtener evidencias de confiabilidad de la prueba para evaluar la percepción de los contrastes mínimos en Lengua de Señas Chilena (LSCh).

**Método:**

Participaron 10 niños y adolescentes Sordos con edades entre los 7 y 14 años, que fueron evaluados con la prueba de percepción de los contrastes mínimos en LSCh. En un primer momento se les aplicó la prueba, y entre 11 y 14 días después se les reaplicó nuevamente (confiabilidad test - retest). Para analizar los datos, fue realizada la correlación Rho de Spearman. Durante la primera aplicación se solicitó autorización a los padres de los niños y adolescentes para grabar las respuestas de los participantes para que otro evaluador pudiese repuntuar los protocolos, con el fin de obtener la confiabilidad interevaluador. Para el análisis de los datos se utilizó el cálculo *estadístico first-order agreement coefficient* (AC1) de Gwet.

**Resultados:**

En la confiabilidad test - retest se obtuvo una correlación fuerte y significativa (Rho= 0,741; p=0,014). En la confiabilidad interevaluador, los valores de concordancia obtenidos varían entre 0,962 a 1 (p<0,001), indicando que la prueba presenta concordancia casi perfecta.

**Conclusión:**

La prueba de percepción de pares de mínimos en LSCh presenta confiabilidad test - retest e interevaluador satisfactoria.

## INTRODUCCIÓN

Las personas Sordas son una población heterogénea^(1)^ tanto niños y adolescentes presentan variabilidad en relación con la proficiencia de la lengua de señas^(2)^. Pues la lengua de señas, muchas veces no es la primera lengua a la cual el niño o niña está expuesto, lo que supone un gran desafío cuando se trata de evaluación. Aproximadamente, el 90-95% de los niños sordos tienen padres oyentes y que desconocen la lengua de señas, por esto muchas veces se atrasa el desarrollo lingüístico del niño, pues la prioridad es la adquisición del habla^(3)^. Por otro lado, los niños Sordos hijos de padres Sordos, presentan contacto con la lengua de señas desde el nacimiento y por ende mayor posibilidad de adquisición de la lengua de señas desde temprana edad.

Durante los últimos años ha habido gran interés por parte de los investigadores en la creación de instrumentos que miden el desarrollo del lenguaje visual en niños sordos^(4)^. La evaluación del lenguaje de un niño o niña requiere de medidas adecuadas que sean apropiadas a su edad, idioma y la cultura^(5)^. Existen pocas evaluaciones que sean válidas y confiables disponibles para los niños sordos, especialmente, las que evalúan el lenguaje entre niños sordos en sus primeros 5 años de vida^(6,7)^. Además se enlistan un escaso número de evaluaciones disponibles para medir las habilidades de desarrollo de la lengua de señas en niños en edad preescolar^(4)^. Lo anterior se contrapone con el interés creciente en la evaluación de los trastornos del lenguaje en niños Sordos que utilizan la lengua de señas como primera lengua^(4,8)^. Gran parte de los instrumentos de evaluación del lenguaje elaborados para personas Sordas son adaptaciones de instrumentos hechos para evaluar el lenguaje en personas oyentes. De esa forma, no reflejan las reales necesidades de la persona Sorda^(7)^. Es posible identificar algunas razones para la escasez de esos instrumentos, como la heterogeneidad de la población y de la lengua de señas, y el bajo acceso a Sordos nativos^(9)^.

Actualmente, en la realidad Chilena son utilizados instrumentos de evaluación construidos para personas oyentes, para evaluar a personas Sordas. Por esto, se requiere de la construcción de pruebas adecuadas para evaluar el lenguaje en la modalidad lengua de señas (visuoespacial).

Existen tests que cuentan con propiedades psicométricas pero estos se encuentran en otras lenguas no en español, por ejemplo: *British Sign Language Vocabulary Tasks* ^(10)^; *American Sign Language Sentence Repetition Test* ^(11)^; *The Visual Communication and Sign Language Checklist* ^(12)^ *.* Además en la Lengua de señas Brasileña (LIBRAS), se elaboró un instrumento para evaluar el dominio de los contrastes mínimos^(13)^ el cual sirve para conocer el desempeño de niños sordos en el reconocimiento y percepción de pares de mínimo contraste en LIBRAS. Además, se estudió el desarrollo de la percepción de los contrastes mínimos en LIBRAS en hijos oyentes de padres Sordos^(14)^.

La evaluación de los contrastes mínimos permite conocer cuál es la percepción que tienen los niños y adolescentes de los distintos parámetros formacionales de la lengua. Además, permite verificar algunos aspectos del desarrollo de esos parámetros en niños^(13)^, que pueden influenciar en la comunicación como un todo. Como la importancia del tiempo de exposición a la lengua de señas, pues cobra sentido en la evaluación de la percepción de los contrastes mínimos en LIBRAS, pues a mayor tiempo de exposición a la lengua de señas, mejor el desempeño en la percepción de los contrastes mínimos^(14)^.

La prueba de evaluación de la percepción de los contrastes mínimos fue adaptada para la Lengua de Señas Chilena (LSCh)^(15)^ y se basa en Vargas et al.^(13)^. Consta de 24 ítems distribuidos de la siguiente forma 7 de configuración manual, 5 de lugar, 8 de movimiento y 4 de orientación. Entre los 24 pares, se seleccionaron 2 ítems como ejemplo (enero-febrero y joven-sufrir). La evaluación pasó correctamente por un proceso de validación de contenido en 7 etapas: 1. Levantamiento de pares de mínimo contraste en LSCh; 2. Análisis de jueces especialistas (dos personas Sordas nativas y dos personas oyentes intérpretes de LSCh); 3. Diseño de los pares de mínimo contraste; 4. Análisis de jueces no especialistas (seis participantes sordos entre 7 y 14 años); 5. Preparación del test; 6. Grabación de video; 7. Estudio Piloto. Estas etapas permitieron presentar una validez de contenido satisfactoria.

A pesar de ser un avance sustancial y convertirse en la primera prueba de evaluación de los contrastes mínimos en lengua de señas Chilena y contar con buenos índices de validez, es necesario hacer más estudios psicométricos, dentro de ellos destaca la confiabilidad. A partir de eso, el objetivo de esta investigación es obtener evidencias de confiabilidad de la prueba de percepción de los contrastes mínimos en LSCh.

## METODO

El presente estudio se encuentra vinculado a un proyecto de investigación debidamente registrado y aprobado por el Comité de Ética en Pesquisa de la Universidad Federal de Santa María, RS, Brasil, folio número 3.022.041. Del mismo modo fue solicitada la autorización de los responsables de los niños y adolescentes que participaron en la investigación, conforme a las normas determinadas por el Consejo Nacional de Salud en su resolución 510/16 mediante la firma de un Término de Consentimiento Libre y Clarificado (TCLE).

### Participantes

Participaron 10 niños y adolescentes Sordos que asisten a escuela especial para personas Sordas y que no poseen ningún otro diagnóstico. Cuatro niños y 6 niñas, con edades entre los 7 y los 14 años al momento de la evaluación (M=11,6 años). Los participantes fueron reclutados por un muestreo no probabilístico por conveniencia, siendo todos de la ciudad de Talca de Chile. Los criterios de inclusión de esta investigación para los niños y adolescentes fueron: ser usuario de LSCh, tener entre 6 y 18 años, tener un diagnóstico de hipoacusia bilateral severa o profunda. Los criterios de exclusión fueron: tener algún otro tipo de déficit observable (lesión neurológica, síndromes, déficit visual no corregido). Todos estos datos fueron colectados a través de una entrevista la cual se aplicó a los padres o cuidadores de niños y adolescentes que participan en este estudio. En la entrevista se preguntó por la edad de exposición a la lengua de señas por parte de los niños y adolescentes, y el promedio de la muestra fue de 5 años. Finalmente, dos evaluadores fonoaudiólogos, intérpretes de LSCh reconocidos por el ministerio de educación, fueron reclutados para análisis interevaluadores.

### Confiabilidad test - retest

Para establecer la confiabilidad test-retest se aplicó de forma completa la prueba de percepción de los contrastes mínimos en LSCh a 10 niños y adolescentes Sordos en dos momentos distintos. El tiempo entre el test y el retest varió entre 11 y 14 días.

La aplicación de la prueba es sencilla, pues, un evaluador encargado le muestra el cuadernillo que contiene las imágenes de los pares de mínimo contraste a los niños y adolescentes Sordos. Además, les muestra un video donde son entregadas las instrucciones en LSCh por un intérprete. El niño (a) u adolescente Sordo debe observar el video, comprender las instrucciones entregadas y responder cuál es el par de mínimo contraste que representó el intérprete, indicando con su dedo en el cuadernillo la alternativa elegida. Si responde correctamente se le asigna el puntaje 1 y si responde de forma errada obtiene 0.

Para análisis de los datos fue utilizado el coeficiente de correlación Rho de Spearman, correlacionando el puntaje total del test con el del retest. Los valores entre 0,31 y 0,5 fueron considerados débiles; entre 0,51 y 0,7 moderadas; entre 0,71 y 0,9 fuertes, y mayores que 0,9 muy fuertes^(16)^.

### Confiabilidad interevaluador

Durante la primera aplicación de la prueba de percepción de los contrastes mínimos en LSCh a los 10 niños y adolescentes Sordos, se solicitó autorización a los padres para grabar el procedimiento. Para esto se utilizó una cámara profesional. Específicamente se grabaron las respuestas de los 10 niños y adolescentes Sordos. Se grabó el momento en que apuntaban la respuesta en la hoja y después de un mes, otro evaluador reanalizó los videos y repuntuó los protocolos. Para verificar la concordancia entre los jueces fue considerado el cálculo *estadístico first-order agreement coefficient* (AC1) de Gwet^(17)^.

## RESULTADOS

La Tabla 1 presenta los datos descriptivos obtenidos en el análisis test-retest considerando el parámetro formacional de los ítems y el test de correlación.

**Tabla 1:** Análisis de correlación test - retest

**Item**	**Parámetro formacional**	**Test M(DE)**	**Retest M(DE)**	**Rho**	**p-value**
Queso - celebrar	Configuración Manual/6	5,6 (0,70)	5,5 (0,71)	0,741	0,014
Miércoles - viernes					
Tortuga - Caracol					
Vaca - toro					
Árbol - Diciembre					
Pato - Pollo					
Cuaderno - Libro	Movimiento/7	5,2 (1,40)	6,6 (0,70)		
Abeja - Mosca					
Mochila - Chaqueta					
Lápiz - Pistola					
Llama - Jirafa					
Cerca - Lejos					
Caminar - Saltar					
Ejemplo - lengua de señas	Orientación/ 4	3,4 (0,97)	3,7 (0,48)		
Jueves - Jugar					
Cómo? - Ocupado					
Positivo - Negativo					
Bonito - Hijo	Lugar/5	3,7 (1,25)	4,6 (0,70)		
Lavar - Acariciar					
Mantequilla - Pintar					
Naranja - fruta					
Amarillo - Verde					

**Subtítulo:** M = Media; DE = Desviación estándar.

Los resultados de la confiabilidad test - retest, del coeficiente de correlación fueron fuertes, positivos y significativos (Tabla 1). Se observa una mejor puntuación de los niños en el retest. Los resultados de confiabilidad interevaluador para cada parámetro formacional son presentados en la Tabla 2.

**Tabla 2:** Resultados confiabilidad interevaluador

**Parámetro formacional**	**Gwet's AC1**	**p-value**
Configuración Manual/6	0,962	<0,001
Movimiento/7	0,977	<0,001
Orientación/ 4	0,966	<0,001

En la Tabla 2 se observa que los valores de concordancia obtenidos varían entre 0,962 a 1 (p<0,001), indicando que la prueba presenta concordancia casi perfecta.

## DISCUSIÓN

La evaluación del lenguaje en la modalidad visuoespacial (lengua de señas), se ha transformado en un verdadero desafío para los investigadores, evaluadores y clínicos^(1)^. Pues son escasas las pruebas que cuentan con propiedades psicométricas (confiables y válidas) para evaluar a los niños Sordos que se comunican por lengua de señas^(7)^. A partir de eso, el objetivo de esta investigación fue obtener evidencias de confiabilidad de la prueba de percepción de los contrastes mínimos en la LSCh.

En la presente investigación se observó resultados satisfactorios de confiabilidad para el instrumento para Evaluar la Percepción de los Contrastes Mínimos en Lengua de Señas Chilena. La confiabilidad es uno de los principales criterios de calidad de un instrumento, pues significa que es capaz de reproducir un resultado de forma estable en el tiempo o a partir de diferentes examinadores^(18,19)^. Del mismo modo el instrumento logró pasar por un proceso de adaptación y validación riguroso, donde se consideraron las características y diferencias lingüísticas y culturales para la adaptación a la LSCh^(20)^.

La confiabilidad test-retest presentó una fuerte asociación entre los resultados del test y los del retest, lo que demuestra la estabilidad temporal del test^(19,21)^. Asimismo, se observa un mejor desempeño de los niños en los retests. Tal resultado puede estar relacionado con el aprendizaje/memoria, ya que el retest se realizó en un corto periodo de tiempo^(18)^, o incluso con la propia adquisición del parámetro formacional. El análisis interevaluador (Tabla 2), evidencia una concordancia casi perfecta, indicando precisión cuando implica a diferentes examinadores de un mismo sujeto.

Sin duda, los resultados obtenidos en la presente investigación son un aporte a la comunidad Sorda Chilena, pues se transforma en el primer instrumento para evaluar los contrastes mínimos en modalidad visuoespacial (lengua de señas) con validez y confiabilidad. No obstante, son necesarios más estudios de validez de constructo, de criterio, de sensibilidad y especificidad.

Entender cómo los niños y adolescentes Sordos perciben los contrastes mínimos puede ayudar en aspectos como entendimiento por parte del clínico de cómo ellos están adquiriendo, comprendiendo y ejecutando las señas. La percepción de los contrastes mínimos que cambian la composición de una seña es importante para la adquisición de la lengua de señas^(13)^. Por lo tanto, el instrumento aquí investigado es de suma importancia para las personas que investigan con esta población.

Como limitación de esta investigación se plantea que el bajo número de participantes se debe a que fue realizada en el contexto de pandemia por COVID-19 y las condiciones sanitarias en Chile al momento de la realización se encontraban en niveles críticos. Es por esto que se recomienda en futuros estudios, ampliar la muestra tanto de niños y adolescentes Sordos como de jueces.

## CONCLUSIÓN

La prueba para evaluar la percepción de los contrastes mínimos en LSCh, cuenta con buenos índices de confiabilidad test - retest y es precisa para el análisis implicando diferentes evaluadores, presentando confiabilidad interevaluador satisfactoria. Es un instrumento replicable y consistente, esto es, que la reaplicación en un mismo sujeto produce resultados similares.
